# Sofosbuvir based therapy in hepatitis C patients with and without cirrhosis: Is there difference?

**DOI:** 10.12669/pjms.331.12163

**Published:** 2017

**Authors:** Shahid Sarwar, Anwaar A. Khan

**Affiliations:** 1 Dr. Shahid Sarwar, MBBS, FCPS (Med), FCPS (Gastroenterology), MCPS-HPE, Associate Professor of Medicine, Services Institute of Medical Sciences, Lahore, Pakistan. Department of Gastroenterology, Doctors Hospital and Medical Center (DH&MC), Lahore, Pakistan; 2 Dr. Anwaar A. Khan, ABIM, ABIM (GE), MACP, FACG, FRCP, AGAF, FCPS. Ex-Dean and Chairman Shaikh Zayed Hospital, Consultant Gastroenterologist, Doctors Hospital and Medical Center, Department of Gastroenterology, Doctors Hospital and Medical Center (DH&MC), Lahore, Pakistan

**Keywords:** Chronic hepatitis C, Cirrhosis of liver, Sofosbuvir, Sustained viral response

## Abstract

**Objective:**

To compare sustained viral response to sofosbuvir/ribavirin ±interferon therapy in patients of hepatitis C with and without liver cirrhosis.

**Methods:**

This observational study of chronic hepatitis C patients was carried out at Doctors Hospital and Medical Center (DH&MC). After diagnostic workup, Sofosbuvir/ribavirin for 24 weeks or sofosbuvir/ribavirin/pegylated interferon for 12 weeks were prescribed. Primary outcome was negative HCV RNA by PCR 12 weeks after treatment completion (SVR_12_). Chi square χ^2^ and student’s t test were used to analyze data.

**Results:**

Of 216 patients included, liver cirrhosis was present in 112 (51.9%) patients and 69(31.9%) were treatment experienced. Liver disease was decompensated in 37 (17.1%) patients. Of 206 patient who completed study protocol, 173(83.1%) achieved SVR_12_, 89.2% (25/28) with triple therapy and 82.2% (148/180) with sofosbuvir/ribavirin therapy. Treatment response was similar between treatment naïve 86.2% (119/138) and treatment experienced 79.4% (54/68) patents. (p value 0.19) SVR_12_ was inferior in cirrhosis patients 75.4% (80/106) as compared to those with no cirrhosis 93% (93/100) (p value < 0.000). It was even lesser in those with decompensated liver disease 68.8% (24/35) (p value < 0.000).

**Conclusion:**

Treatment outcome with sofosbuvir/ribavirin combination therapy in cirrhosis patients is suboptimal especially in those with decompensation as compared to patients without liver cirrhosis.

## INTRODUCTION

Hepatitis C is among the leading causes of health related morbidity and mortality with ever rising incidence.^1^ Due to its dreaded complications resulting in repeated hospitalization and need for liver transplantation, it consumes a major share of health related resources.^2^

Interferon based therapy was the only treatment option until few years back with its troublesome side effects and inadequate success rate. Introduction of directly acting antiviral (DAA) drugs has revolutionized management of hepatitis C.^3^ Sofosbuvir was the first nucleotide analogue which was effective without the need for interferon treatment.^4^ It also opened the gateway for so more DAAs with excellent treatment outcome.^5^

With increasing use of Sofosbuvir based treatment for hepatitis C, difficult to treat aspects of hepatitis C patients are being recognized. Genotype 3 (G-3) is emerging as difficult to treat and very few drugs are effective for its treatment.^6^ Response rate for these patients with all oral therapy is at best 80-85%, much inferior to genotype 1 or 2.^7^ Treatment options for G-3 are not satisfactory, especially for those who are treatment experienced or have already developed cirrhosis. Arias et al concluded that presence of advanced fibrosis predicts treatment failure.^8^

Predominant genotype in Pakistan is 3^9^ and with Sofosbuvir, the only available DAA we needed to explore the outcome of this treatment in our patients especially those with advanced liver fibrosis. We planned a study to compare outcome of Sofosbuvir based therapy in terms of sustained viral response at 12 (SVR12) in patients with chronic hepatitis C with and without liver cirrhosis.

## METHODS

This observational study was carried out at Hepatology Clinic at Doctors Hospital & Medical Center (DHMC) from October 2014 to September 2016. Only confirmed cases of chronic hepatitis C as determined by positive polymerase chain reaction (PCR) with lower limit of detection of 10 IU/ml were included after informed consent. All patients had complete blood count, clotting profile, liver function tests, renal profile and abdominal ultrasound examination. Patients with evidence of cirrhosis on ultrasound abdomen i.e. coarse texture, shrunken liver, splenomegaly or presence of ascites and those with advanced fibrosis (F3 and F4) on shear wave elastography were labeled as “cirrhosis patients”. Child Pugh Turcotte (CTP) score was used to stage cirrhosis patients.

Patients with uncontrolled diabetes mellitus, hypertension, unstable cardiac failure, stroke or any major co-morbid illness were excluded. Patients with hemoglobin less than 10 g/dl, platelet count of 30,000/mm^3^ or less, alanine aminotransferase, aspartate aminotransferase or alkaline phosphatase, bilirubin values 10 times or more than the upper normal limit, and creatinine clearance less than 30ml/min were also excluded.

Patients with genotype 2 were treated with sofosbuvir and ribavirin for 12 weeks if no cirrhosis and 24 weeks for cirrhosis patients. Genotype 1, 3 and 4 patients were offered both available options of pegylated interferon, sofosbuvir and ribavirin for 12 weeks or sofosbuvir and ribavirin for 24 weeks with expected response rate and side effect profile. Dose of sofosbuvir was 400 mg daily while that of ribavirin was 1000mg and 1200mg for patient weighting < 75kg and ≥75kg respectively while pegylated interferon alpha 2a was prescribed 180µg weekly while dose for alpha 2b was 1.5µg/kg/week.

Patients were followed monthly with complete blood count, liver function tests and renal profile. Patients with negative HCV RNA by PCR at end of treatment and 12 weeks later were considered to have sustained viral response (SVR_12_), those with positive HCV RNA at end of treatment were non-responders whereas those with negative PCR at end of treatment but positive test after 12 weeks were labeled as relapsers.

### Statistical Analysis

SPSS 20^®^ was used for statistical analysis. Numerical variables were given as mean ± standard deviation (SD) or median whereas, nominal and categorical variables were given as percentages. Unpaired student’s t test was used to compare numerical variables whereas, chi square (χ^2^) was used for categorical or nominal variables between patients with and without cirrhosis of liver. Data were analyzed per protocol and P value of < 0.05 was considered significant.

## RESULTS

Total of 216 patients were included in the study. Mean age was 49.4 (±12.1) years with male to female ratio of 1.1 (114/102). Liver disease was already de-compensated in 37 (17.1%) patients, 22 (10.2%) had variceal bleeding, 21(9.7%) ascites whereas 3 had history of encephalopathy. Diabetes mellitus was present in 31 (14.4%) patients and 26 (12%) were hypertensive. Treatment naïve patients were 147(68.1%) and 69 (31.9%) were treatment experienced patients. Genotype 3 was the predominant genotype, 206(95.4%) patients, 8 (3.7%) patients had genotype 1 and 1 patient each of genotype 4 and 6 were included. Liver cirrhosis as determined by abdominal imaging and fibroscan was present in 112 (51.9%) patients while three (1.4%) patients had hepatocellular carcinoma. Of 112 patients with cirrhosis, 76 (67.8%) had CTP class A, 34 (30.3%) class B and two (1.9%) patients were of class C. Patients with and without cirrhosis are compared in table I.

**Table-I:** Comparison of patients with and those without cirrhosis of liver.

Variables	No cirrhosis (n-104) (mean±SD)	Cirrhosis (n-112) (mean±SD)	P value
Age (years)	46.2 (±13.3)	52.3 (±10.26)	<0.00
Platelet count (x 10^9^/L)	232 (±73.4)	122.9 (±54.6)	<0.00
INR	0.9 (±0.05)	1.18 (±0.26)	<0.00
Serum albumin (g/dl)	4.1 (±0.37)	3.42(±0.55)	< 0.00
CTP score	5.08(±0.3)	6.19(±1.3)	<0.00
Diabetes mellitus (No of patients)	19	120.27	
Treatment experienced patients	29	400.32	

SD: Standard Deviation, INR: International Normalization Ratio, CTP: Child Turcotte Pugh

Sofosbuvir/ribavirin was started in 188 (87%) patients while 28 (13%) patients received pegylated interferon, sofosbuvir and ribavirin. Major side effects experienced were fatigue 110(50.9%), headache 25(11.6%) and fever 24(11.4%). Worsening of ascites was noted in 10 (4.6%) patients, worsening encephalopathy in 6(2.8%) patients, 2 of whom died whereas three(1.4%) patients had variceal bleeding during treatment. Majority of patients had decline in hemoglobin with 58(26.9%) experiencing drop of more than 3 g/dl which was managed with ribavirin dose adjustment, erythropoietin injection and iron supplementation, where needed.

Treatment was completed by 208 (96.3%) patients, five (2.3%) stopped follow up during treatment and treatment had to be discontinued in three (1.4%) patients, one developed pulmonary tuberculosis whereas, two patients died due to acute on chronic liver failure during treatment. Of 208 patients with complete follow up 194 (93.4%) had negative HCV RNA at end of treatment whereas, 14 (6.6%) were non-responders. SVR_12_ was achieved by 173 (83.18%) patients, 19 (9.14%) patients had relapse while two more patients were lost to follow up. Patients who achieved SVR_12_ and those who failed in attaining sustained response are compared in table II.

**Table-II:** Comparison of patients with SVR12 and those who failed to achieve SVR12.

Variables	Patients with SVR12 (n-173) Mean(±SD)	NO SVR12 (n- 36) Mean(±SD)	P value
Age (years)	49.2 (±12.5)	50 (±11)	0.53
Duration of illness (months)	50.6 (±48.2)	58.3 (±58.4)	0.58□
Platelet count (x 10^9^/L)	182.7 (±86)	143.5 (±69.7)	0.50□
INR	1.06 (±0.13)	1.24 (±0.4)	0.32□
Serum bilirubin (mg/dl)	0.83 (±0.54)	1.16 (±0.99)	0.83□
Serum albumin (g/dl)	3.82 (±0.58)	3.45 (±0.58)	0.001
No of patients with cirrhosis	80	29	<0.000
CTP score	5.54 (±0.9)	6.28 (±1.52)	<0.000

INR: International normalization ratio, CTP: Child Turcotte Pugh, □ Mann Whitney U test

SVR_12_ with pegylated interferon based triple therapy was 89.2% (25/28) whereas, it was 82.2% (148/180) with sofosbuvir/ribavirin therapy and difference was not significant (p 0.32). Similarly treatment naïve patients had 86.2% (119/138) SVR_12_ whereas, it was 79.4% (54/68) for treatment experienced patients and difference was non-significant (p value 0.19). All 6 patients with genotype 1 who completed treatment, achieved SVR_12_ as did one patient each with genotype 4 and 6. Overall SVR_12_ for genotype 3 was 83.3% (165/198)

Of 106 patients with cirrhosis, 80 (75.4%) had SVR_12_, 12 (11.3%) were non-responders, 14 (13.3%) had relapse. Of 100 patients with no cirrhosis, completed study protocol, 93 (93%) had SVR_12_, two (2%) were non-responders and five (5%) had relapse. Patients with cirrhosis of liver had significantly inferior response to treatment as compared with patients without cirrhosis (p value <0.000). Treatment response was even lesser with decompensated liver disease wherin, SVR12 was 68.6% (24/35) as compared with 87.1% (149/171) in those without decompensated liver disease (p value 0.006). On comparing SVR_12_ among different stages of cirrhosis, it was 75.6% (56/74) for CTP A and 75% (24/32) for CTP B and C.

## DISCUSSION

Patients with hepatitis C and cirrhosis of liver are among priority candidates, for treatment as per AASLD guidelines.^10^ In a study by Deterging K. et al, which included 43% patients with CTP class B and C, improvement in MELD score was noted in 44% patients.^11^ Foster GR et al noted mean reduction in MELD score of 0.85 in patients with decompensated cirrhosis treated with nucleotide analogues.^12^ Viral eradication results in reduced HCV related complications and gives opportunity to perform liver transplantation in virally cured patient from HCV.^13^

SVR_12_ was achieved in 89.2% of our patients with triple therapy and in 82.2% patients with sofosbuvir/ribavirin therapy. In BOSON study by Foster GR et al, SVR_12_ for genotype 3 patients was 84% for sofosbuvir/ribavirin and 93% for pegylated Interferon/sofosbuvir/ribavirin therapy.^14^ Similarly response rate in patients of genotype 3 was 83% in study group of Lawitz E et al.^15^

We have noted significantly inferior SVR_12_ (75.4%) in patients with cirrhosis of liver as compared to those with no cirrhosis (SVR_12_ 93%). In TARGET study by Feld JJ et al of genotype 3, viral clearance was achieved in 58% of treatment naïve patients with cirrhosis while those with cirrhosis and treatment experienced, SVR_12_ was 42%.^16^ Jacobson IM et al, in a study of genotype two and three patients, noted lower response rate in genotype 3 and even lesser in cirrhosis of liver.^17^ In a study of 419 patients, Zeuzem S et al noted 85% SVR_12_ with sofosbuvir/Ribavirin, 91% for patients without cirrhosis while 68% in patients with cirrhosis in genotype 3, fairly close to what we have noted in our patients.^18^

SVR_12_ is not different in our study for treatment naïve and treatment experienced patients as was observed in TARGET study.^16^ Among patients with cirrhosis, it is the presence of decompensated liver disease which further reduces the chances of having SVR_12_ as it was 68.6% in patients with decompensated cirrhosis in our study. Response rate noted by Foster GR et al, in 409 patients with decompensated cirrhosis due to genotype 3, was 68.8% with sofosbuvir/Ribavirin therapy.^12^ Low serum albumin, high CTP score and presence of cirrhosis were associated with less chances of SVR_12_ in our patients as shown in Table-II. Decompensated cirrhosis patients who do achieve SVR with treatment have improvement in their liver functions with better quality of life.^19^

In view of suboptimal response with sofosbuvir/Ribavirin therapy in genotype 3 patients with cirrhosis, newer drugs like Daclatasvir^20^ and recently approved combination of sofosbuvir and valpatasvir,^21^ should be made available, which have shown much better results for genotype 3 patients even with advanced liver disease.

Majority of our cirrhosis patients were of CTP class A and B whereas only two patients with class C were included. Treatment of CTP class C patients is more challenging with likelihood of poorer results and more side effects.^16^ Further studies of this difficult to treat population are needed to determine merits of treatment and its potential risk in patients of advanced liver disease.

## CONCLUSION

Treatment outcome with sofosbuvir/ribavirin combination therapy in genotype 3 patients with cirrhosis is suboptimal especially in those with decompensated disease as compared with patients without liver cirrhosis.
